# Sacrificial Lambs and Rallying Cries: The Politics of Adult Education at the University of Manitoba, 1907–1949

**DOI:** 10.1177/07417136241308219

**Published:** 2025-02-10

**Authors:** Scott McLean

**Affiliations:** 1 Department of Sociology, 192287 University of Calgary, Calgary, AB, Canada

**Keywords:** politics of adult education, history, university extension, Canada

## Abstract

This article narrates the engagement of the University of Manitoba in two waves of the extension movement that shaped adult education work at universities across North America: one rooted in the delivery of public lectures and another rooted in the “Wisconsin idea” of serving citizens and the state. In contrast to developments at provincial universities to the west and east, extension work at the University of Manitoba was not institutionalized until 1949. The article analyzes the politics of adult education at the University of Manitoba, arguing that extension work was treated at different times as a sacrificial lamb and as a rallying cry by university administrators in relations with the provincial government. It analyzes economic and institutional factors that explain why the politics of adult education were so different in Manitoba than in nearby provinces. It offers insight for those interested in adult education programs delivered through universities beyond Canada.

## Introduction

Around the turn of the twentieth century, adult education gained prominence at universities across North America through two waves of an extension movement—each characterized by a distinctive philosophical ethos and programmatic focus. In the early 1890s, university leaders established the American Society for the Extension of University Teaching (ASEUT) and the Canadian University Extension Association (CUEA). Both organizations positioned university extension as a movement dedicated to the democratization of higher education, and both promoted off-campus lectures as the means through which such democratization would be accomplished. George James, ASEUT General Secretary, wrote that university extension was “the bringing of the university to the people” such that the “privileges of knowledge shall be no longer only for those who are able to satisfy the conditions of academic residence” (James, 1891, p. 55). In announcing the establishment of the CUEA, its leaders stated that the aim of the organization was “to bring within reach of the people opportunities of sharing in the benefits of higher education” (CUEA, 1892, p. 2). Both organizations attracted leaders of prominent universities as members and advocated for universities to provide off-campus lectures, grouped into courses, which would lead to certificates for students successfully completing examinations at the end of those courses. While the systematic provision of courses, examinations, and certificates was never accomplished in Canada (MacLaughlin, 1894) and lasted only a few years in the United States (Van Hise, 1915), this early extension movement did foster the engagement of universities in the provision of public lectures for adults not enrolled as students.

In the early 1900s, Charles Van Hise—President of the University of Wisconsin—repositioned university extension within an ethos of “service” to be provided “directly to the people of the state and the nation” (Van Hise, 1915, p. 8). Van Hise argued that through extension service, “the university shall carry to the people the knowledge which they can assimilate for their betterment along all lines” (Van Hise, 1915, p. 8). He criticized the “lyceum method” of previous university extension work and described the diverse forms that such work should take: courses delivered by correspondence or at off-campus locations; applied research and information dissemination; technical services provided to state and municipal governments; the organization of debates and public discussion; and the provision of educational exhibits and presentations at fairs, institutes, and conventions (Van Hise, 1915). Van Hise's vision of university extension migrated to Canada with the establishment of public universities in the provinces of Saskatchewan and Alberta. Following a study tour in Wisconsin, Walter Murray, first president of the University of Saskatchewan, wrote:

What is the sphere of the university? Its watchword is service—service of the state in the things that make for happiness and virtue as well as the things that make for wealth. No form of that service is too mean or too exalted for the university. It is as fitting for the university, through correspondence classes, extension courses, supervision of farmers’ clubs, traveling libraries, women's institutes or musical tests to place within the reach of the solitary student, the distant townsman, the farmer in his hours of leisure or the mothers and daughters in the home the opportunities for adding to their stores of knowledge and enjoyment, as it is that the university should foster researches into the properties of radium or the causes and cure of swamp fever. (University of Saskatchewan, 1909, pp. 11–12)

By 1910, the “Wisconsin idea” had been adopted in Saskatchewan and Alberta, and the delivery of public lectures by professors had become common practice at universities in Ontario.

This study contributes to the history of the extension movement at universities in North America by examining the case of a university in which neither wave of that movement became institutionalized in the first half of the twentieth century. The University of Manitoba was established as a federation of three denominational colleges in 1877 and became a publicly funded, provincial university in 1917. Despite being surrounded (with Saskatchewan, Alberta, and British Columbia to the west, Toronto to the east, and Minnesota and Wisconsin to the south) by universities with major extension operations and despite repeated efforts by its leaders, the University of Manitoba did not receive public funding for extension work until 1949. This article examines the politics of adult education by narrating the history of unsuccessful efforts to institutionalize extension work at the University of Manitoba and contrasting that history with experiences at the universities of Toronto, Saskatchewan, Alberta, and British Columbia. Grounded primarily in archival research, the paper also employs official statistics from five Canadian provinces to explain why the politics of adult education were so different at the University of Manitoba than at neighboring institutions.

The development of extension at the University of Manitoba was part of a broader history of adult education in North America. Readers wishing to situate this study within the historical context of adult education in North America may consult Kidd (1950), Moreland and Goldenstein (1985), Selman (1995), Stubblefield (1988), Stubblefield and Keane (1994), and Welton (2013). Overviews of the university extension movement are available for the United States (Portman, 1978; Shannon & Shoenfeld, 1965; Woytanowitz, 1974) and Canada (Corbett, 1952; Kidd, 1956). Firsthand accounts of both waves of the university extension movement are accessible via the Hathi Trust: see the journal *University Extension* published monthly by the ASEUT from 1891 through 1894 and the proceedings of the *Annual Conference of the National University Extension Association* published from 1915 through 1926. I have previously narrated the history of extension at universities in Quebec, Ontario, Saskatchewan, Alberta, and British Columbia (McLean, 2024, 2023a, 2023b, 2022, 2012, 2011, 2009, 2008, 2007a, 2007b), while Alexander (1997), Webb (2014), and Welton (2001) have done so for Atlantic Canada. While Kops (1996) and Starosilec (1997) have written about the more recent history of adult education at the University of Manitoba, neither examines the period prior to 1949. Welton (1983, 1987, 2013) mentions the University of Manitoba but does not narrate its engagement with adult education.

**Figure 1.**
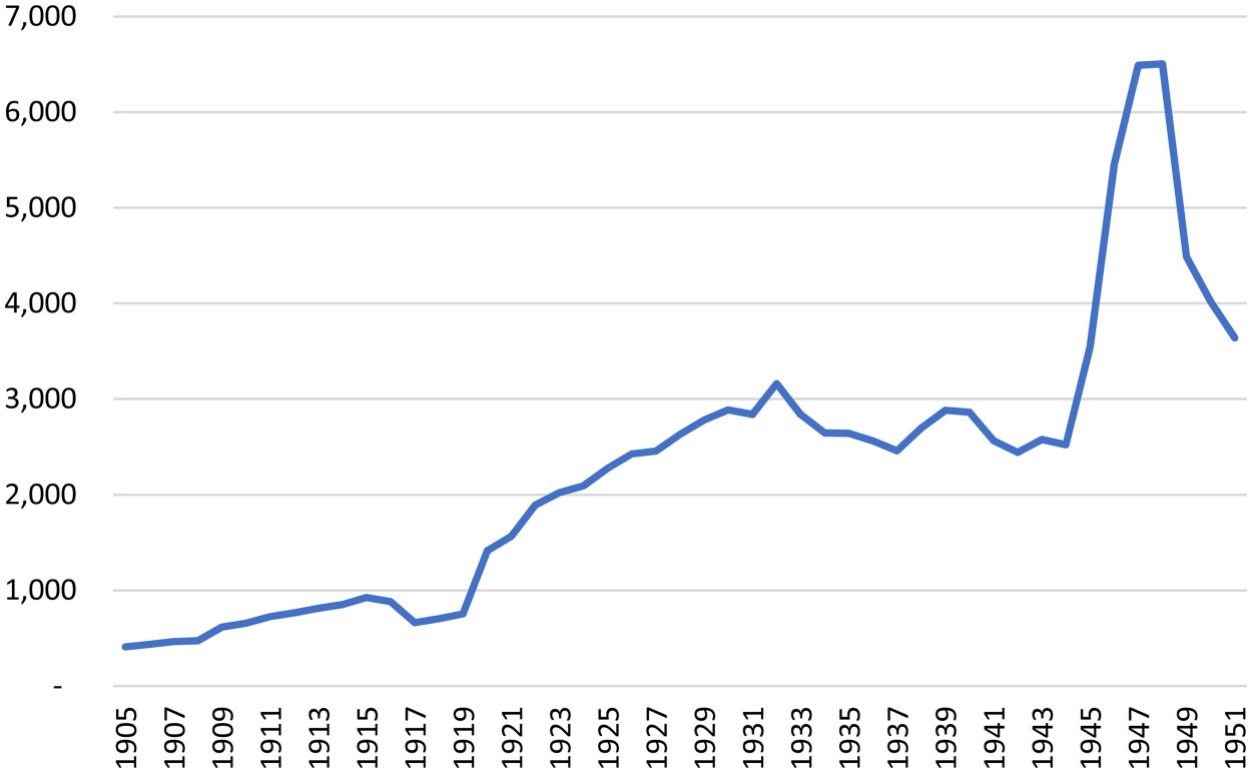
Enrollments at the University of Manitoba, 1905 through 1951.

Source: *Annual Reports of the University of Manitoba*.

**Figure 2.**
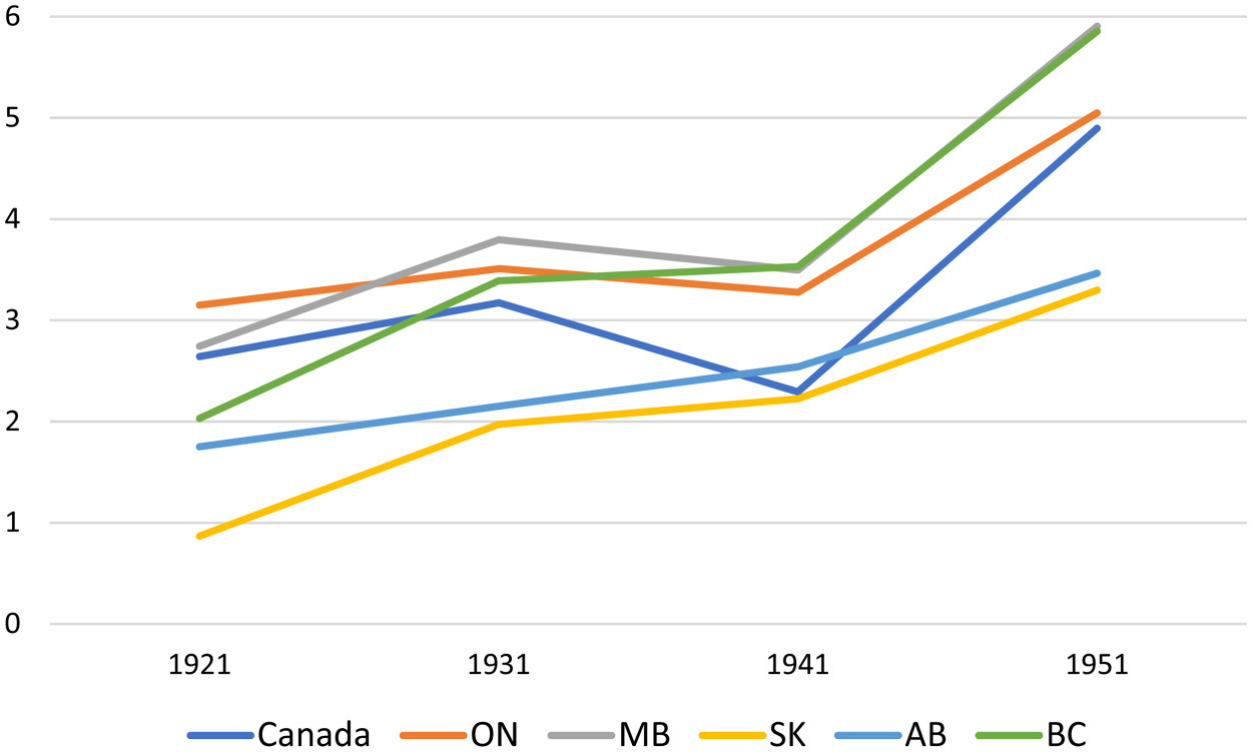
Number of full-time university students per 1,000 residents, 1921 through 1951.

Source: Statistics Canada, 1983: Tables A2–14, W340–438a through W340–438f.

In the 1920s, the University of Manitoba grew to become the second largest university in Canada, enrolling over 2,500 full-time students throughout the 1930s (see Figure 1). However, as was the case across Canada in the first half of the 1900s, relatively few Manitobans attended university. Figure 2 documents the number of full-time university students for each 1,000 residents, for Canada and its five westernmost provinces. To place Figure 2 in perspective, in 2021, there were 38 full-time university students for each 1,000 residents of Canada (Statistics Canada, 2023a, 2023b). Another indicator of how few Canadians attended university in the first half of the twentieth century is that the total number of university students in Canada grew from 23,214 to 68,595 between 1921 and 1951 and subsequently skyrocketed to 1,413,762 by 2021. In the first half of the twentieth century, the politics of adult education at Canadian universities were dominated by the fact that very few Canadians attended those universities as full-time students. As a result, extension work was an important means through which most publicly funded institutions built social legitimacy and political capital.

The University of Manitoba provides an important historical study of the politics of adult education because, despite becoming a relatively large institution, it did not receive public funds for extension work until 1949—decades after provincial universities in Ontario, Saskatchewan, Alberta, and British Columbia had established significant extension departments (note that the University of Toronto functioned as the provincial university of Ontario). Between 1907 and 1917, public lectures and evening courses were cited prominently—as evidence of the social relevance of the institution which in those years received very little public funding—in the annual reports of the university. University President James MacLean (tenure from 1913 through 1934) repeatedly and unsuccessfully tried to convince the provincial government to provide financial support for extension lecture work. University President Sidney Smith (tenure from 1934 through 1944) endeavored to build political capital with the provincial government through delivering a Wisconsin-style extension program funded by user fees and grants from philanthropic organizations. University President Albert Trueman (tenure from 1945 through 1948) allowed the scope of extension work to decline substantially, ironically despite chairing the Manitoba (provincial) Royal Commission on Adult Education in those same years. This article documents the participation of the University of Manitoba in both waves of the extension movement (public lectures and the Wisconsin idea), compares such participation to that of other provincial universities in Ontario and Western Canada, and analyzes comparative socio-economic and institutional factors that explain why the politics of adult education were so different in Manitoba than in nearby provinces.

## Extension Lectures as a Sacrificial Lamb: Seeking Public Funding From 1907 to 1934

In the early 1900s, the University of Manitoba consisted of denominational colleges providing instruction in theology and the liberal arts, along with the Manitoba College of Medicine and the Manitoba Law School (Special Committee, 1937). In 1904, the university appointed its first faculty members to teach courses through a “University Faculty” rather than a denominational college or professional school (Morton, 1957). The six initial appointees were in physics, chemistry, botany/geology, physiology/zoology, bacteriology, and mathematics. Additional departments of instruction were created in 1907 (civil engineering), 1909 (electrical engineering, English, political economy, and history), 1913 (architecture, French, and German), and 1914 (pharmacy and classics). In 1917, the University of Manitoba became a provincial university, financially supported by taxpayers and accountable to government (Morton, 1957). The provincial government began providing significant funding to the university and committed to purchasing a site and building a permanent campus for the institution.

The first efforts to provide educational services to adults not enrolled as regular students at the University of Manitoba were popular lectures delivered on-campus in Winnipeg. This “Annual Series of Popular Lectures” was delivered in February every year beginning in 1907. Each year, four faculty members delivered lectures on successive Friday evenings “with the object of bringing the public into closer touch with the work of the University” (University of Manitoba, 1910, p. 2). By the eighth year of these popular lectures, they were attracting large audiences: “Through these lectures the University of Manitoba is brought prominently before a large number of people of the city, and the interest has now become so general that the largest lecture theatre available in the University Building is entirely unable to accommodate the audiences” (University of Manitoba, 1914, p. 2). Between 1919 and 1934, the university hosted seventy-six such on-campus lectures, whose topics included those related to the sciences (e.g., “Heredity”), classical studies (e.g., “Homer's Odyssey”), current events (e.g., “India in the Empire”), engineering (e.g., “Bridges and Bridge-Building”), and medicine (e.g., “The Craft of Surgery”).

The university's second adult education initiative began in 1909 when professors of English and political economy, having very few full-time undergraduates to teach, delivered courses on evenings and Saturdays to adults employed elsewhere during the work week. In 1909–10, the Department of English delivered a “Saturday Course for Teachers” and an evening lecture series on Thursdays (University of Manitoba, 1910); the Department of Political Economy delivered “two lectures per week on political economy, for those engaged in business and the professions” (University of Manitoba, 1910, p. 20). Professor Clark claimed that the evening course in political economy “appeared to meet a real need, and the lectures were attended by a very earnest, appreciative, and interesting class of men” (University of Manitoba, 1910, p. 20). In subsequent years, several other departments offered evening classes on an irregular basis, including those in history, architecture, geology, and engineering.

The most significant early adult education initiative at the University of Manitoba was the delivery of “extension lectures” at locations outside of Winnipeg. Having the goal of “extending still further the influence of the University throughout the Province” (University of Manitoba, 1912, p. 2), faculty members delivered twenty-one such lectures in 1911–12 and twenty-four in 1912–13 (University of Manitoba, 1912; University of Manitoba, 1913). In 1913, the University of Manitoba established a “Committee on Extension Lecture Work,” and for the subsequent three years, Lloyd Warren, an assistant professor of mathematics, chaired the committee. Warren reported very substantial participation in the first two years. The extension lecture service was a cost-recovery initiative, with local organizations paying a small fee ($25 for three lectures) to offset travel and administrative costs and with faculty members delivering the lectures without financial compensation beyond their regular salaries. Despite a promising start, the extension lecture service ground to a halt in 1915, with Warren citing two constraints: fewer lecturers available given the enlistment of faculty members in the armed forces and financial constraints facing host organizations (University of Manitoba, 1916).

The lecture service was reinvigorated in 1917, through administrative and programmatic changes. Administratively, the University Council established a Council Committee on Extension Work, initially chaired by Professor of Architecture, Arthur Stoughton (University of Manitoba, 1918). Programmatically, Stoughton expanded the number of extension lectures by working in collaboration with external agencies: the Extension Service of the Department of Agriculture, the Social Services Council, the School Trustees Association, the Grain Growers’ Association, and the Retail Merchants’ Association (University of Manitoba, 1918). While the extension lecture service was paused due to the global influenza epidemic in 1918 and the Winnipeg general strike in 1919, the scope of lectures delivered through the University of Manitoba remained substantial throughout the 1920s.

**Figure 3.**
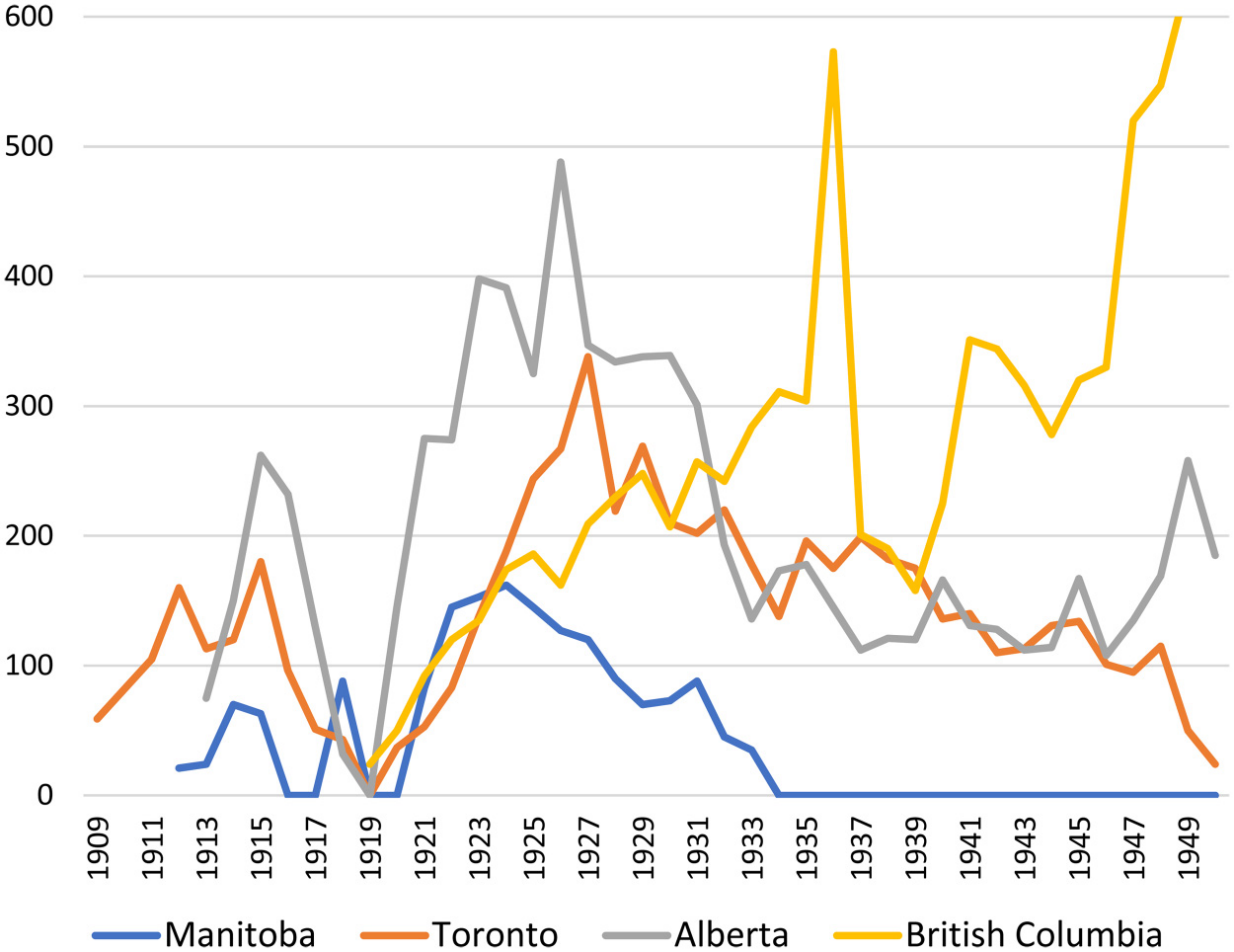
Number of extension lectures delivered by provincial universities, 1909–1950.

Source: Annual Reports of the President from each university.

Figure 3 compares the number of extension lectures delivered by professors from the universities of Toronto, Manitoba, Alberta, and British Columbia. While universities did not consistently report the number of people in attendance at such lectures, annual attendance peaked at over thirty thousand for extension lectures offered through the University of Toronto and the University of Alberta in the latter 1920s and at over forty thousand for those of the University of British Columbia in the latter 1940s. Average annual attendance at extension lectures organized by the University of Manitoba was about 15,000 in the 1920s. Figure 3 shows that while the University of Manitoba developed a significant extension lecture service in the 1910s and 1920s, it delivered far fewer lectures than other provincial universities and it permanently stopped offering extension lectures during the Great Depression. The following analysis of the politics of adult education at the University of Manitoba during this era shows that extension lectures were treated as a sacrificial lamb by university administrators—something valuable and important but ultimately something to be set aside when presented with the opportunity to receive funding for higher priorities.

In 1918, the first Chair of the Board of Governors at the University of Manitoba, Isaac Pitblado, commented on the university's extension lecture work at points outside Winnipeg:

The response of people everywhere was usually cordial, due, it is believed, to their appreciation of the new status of the University as a Provincial institution. The members of the Board are unanimous in the opinion that this department of University work constitutes a most important function of our University, and look to see its activities continually enlarged. (University of Manitoba, 1918, p. 4)

Two years later, Pitblado praised the work of university extension and called upon the provincial government to fund such work:

The extension work of the University both in the form of evening classes and in the form of individual lectures given at local centres throughout the province has been well maintained …Your Board is of the opinion that the demands of this work call for the appointment at an early date of a Director and Assistants who will devote their time and energy exclusively to its prosecution … unless the University assembles the resources necessary to meet this demand and applies them to the task, it will fall short of its privilege and duty as leader of the educational agencies of the province. (University of Manitoba, 1920, p. 5)

In 1919, Norman MacDonald, a Lecturer in History, took over from Stoughton as Chair of the Committee on Extension Work. MacDonald narrated a “democratizing” philosophy of extension that MacLean quoted in his President's Report:

University extension work should, I think, be made available and serviceable to the greatest number of people in Manitoba … The number of students who come to us is small compared to the vast crowd who cannot, for various reasons, avail themselves of all the privileges an intra-mural training can give them; and since so many cannot come to the University, and yet are ambitious to learn, to improve their minds and opportunities, their usefulness to themselves and the province, it seems to me, the University must go to them. (University of Manitoba, 1920, p. 18)

MacLean followed this quotation with the observation that the university required more resources to institutionalize extension work: “it is evident that the demand for this type of University service is widespread and permanent, and has increased in volume to the extent that it requires the services of a Director and a small staff of assistants” (University of Manitoba, 1920, p. 20).

In subsequent years, MacLean and Pitblado regularly asked—in their annual reports to the Minister of Education—the provincial government for additional resources to support extension work. In 1921, MacLean wrote, following a detailed description of extension lectures and evening and short courses offered by the university over the previous year:

It would seem therefore that the effective demand for the extension services of the University is permanent, is increasing, and may be expected to expand. On the other hand it seems to be quite certain that the financial resources of the University do not permit any expansion or multiplication of these services at the present time. It may even be necessary to discontinue for a period some of the courses already undertaken. (University of Manitoba, 1921, p. 16)

Here, one sees MacLean threatening to sacrifice the lamb of extension work if funding were not increased. The following year, Pitblado documented the rapid growth of both “regular” and “extension” enrollments and proceeded to cajole the provincial government into providing more resources for the University of Manitoba:

This growth … represents a substantial advance in our community in the matter of interest in higher education and one that is likely to be sustained and, indeed, to increase greatly in the years to come. It would appear, therefore, that we, as members of the University Board of Governors, and your Government, as custodians of the public revenues of the Province, must address ourselves to the task of providing added financial support for that which the public demand, and which admittedly is for the advantage of the state. (University of Manitoba, 1922, p. 2)

In 1926, Pitblado's successor, John Machray, outlined the ongoing growth of undergraduate enrollments and extension lectures, and wrote, “It is earnestly hoped that in the very near future it will be possible for your Government to make financial provision for certain increases in the organization and staff that will make it possible for your Board to meet at least some of the demands for enlargement of its programme” (University of Manitoba, 1926, p. 4). In 1927, Machray repeated his request for financial support for extension work: “Your Board feels that the demands of this work are now such that it might very well receive the full attention of a member of the staff throughout the entire fall and winter season” (University of Manitoba, 1927, p. 6).

Despite repeated requests from Pitblado, MacLean, and Machray for provincial funding in support of extension work, the provincial government at no point prior to the 1940s provided operational funding dedicated to such activities. In 1928, Machray illustrated the frustration of senior university administrators with this situation:

It is now quite clearly established that rural lecture work cannot be further developed without the full time service and attention of a member of the University staff. It is hoped that the plan your Board has entertained for some time with regard to such an appointment will be realized during the coming year. (University of Manitoba, 1928, pp. 6–7).

In the late 1920s, Machray and MacLean ceased asking the provincial government to support extension work and instead focused attention on the financial commitments required to build a permanent campus for the university. In the early 1930s, Machray was imprisoned for defrauding the University of Manitoba, and MacLean focused his reports on the work of building a permanent campus (University of Manitoba, 1933).

Despite the absence of financial support from the provincial government, from 1927 through 1933 the annual reports of the University of Manitoba included a “Report of the Extension Department.” In mid-1933, the University of Manitoba closed its Extension Department and terminated its extension lecture service. In his final report as president, MacLean noted that “almost every university is extending and improving her programme for popular instruction (University of Manitoba, 1934, p. 5).” but characterized the popular education work of the University of Manitoba as “feeble and inadequate” in comparison to that of the universities of Saskatchewan and Alberta (University of Manitoba, 1934, p. 5). He explained that the “first duty” of the university “was to furnish adequate courses of instruction to the students who came to the University in numbers that increased from year to year” (University of Manitoba, 1934, p. 5). He explained:

An extension programme requires the appointment of a director and extension staff, and the expense of maintenance could not be fitted into our annual budget. Also there is an old adage—“When two steps are to be taken it is better to take the first step first”—and common sense suggested that it was advisable to build at least the frame-work of a university before embarking on elaborate plans for its extension.

This is an interesting passage, since it repeats MacLean's longstanding position that budgetary constraints hindered extension work while introducing, for the first time in his reports, the claim that strategic institutional policies led to a focus on intramural instruction over extension work. This claim was at odds with two facts: the University of Manitoba had engaged in widespread extension work for twenty years; and MacLean, Pitblado, and Machray had repeatedly asked the provincial government to fund extension work. MacLean finished his tenure at the University of Manitoba by positioning adult education as a sacrificial lamb: a valuable activity that could not be properly conducted due to higher priorities and financial constraints—constraints that had become more pronounced due to the Great Depression and the fact that Machray had embezzled and lost virtually all of the university's endowment funds in bad investments (Morton, 1957). Whatever the intention behind MacLean's rhetoric, the outcome was that the University of Manitoba stopped delivering extension lectures at a time when other provincial universities served tens of thousands of adults annually through such work.

## The Wisconsin Idea as a Rallying Cry: Building Political Capital, 1934 to 1949

The philosophical ethos and programmatic focus of adult education at the University of Manitoba shifted in 1934 with the arrival of a new president, Sidney Smith. Smith criticized “the shallowness and superficiality which sometimes characterize so-called extension work” (University of Manitoba, 1935, p. 2) and spearheaded a dramatic expansion and diversification of adult educational programs along the lines of the Wisconsin idea. Such programs were not publicly funded; some relied on tuition fees and others relied on philanthropic donations made by American foundations. Smith deployed Van Hise's rhetoric of extension as service in his annual reports during his decade as president. In his first report, Smith described teaching and research as two key responsibilities of a university, and then argued:

Moreover, a university should strive to benefit directly a larger constituency than its immediate student body; it should be a centre from which rich intellectual and aesthetic influences may emanate for the improvement of the people at large … There is, I believe, an opportunity for the University of Manitoba to reach out beyond its walls and present new intellectual and aesthetic horizons to its shareholders—the taxpayers of Manitoba—whether they live in the metropolitan area of Winnipeg or in the other cities and in the towns and rural communities of this Province (University of Manitoba, 1935, p. 1).

Two years later, Smith argued that the university had a “duty” to “minister directly” to the “needs” of its “shareholders” (University of Manitoba, 1937, p. 3). He used a topographical metaphor to further this message: “the University cannot reside and thrive upon a mountain top, removed from the people on the slopes and in the valley. It derives its support in money and students from the people, and it must keep in contact with them in order that it may serve them the better.” Smith was aware of the need to cultivate political capital with rural politicians: “in this agricultural province, support for the University in the Legislative Assembly depends in no small degree upon the goodwill and interest of representatives of the rural constituencies” (University of Manitoba, 1937, p. 9).

**Figure 4.**
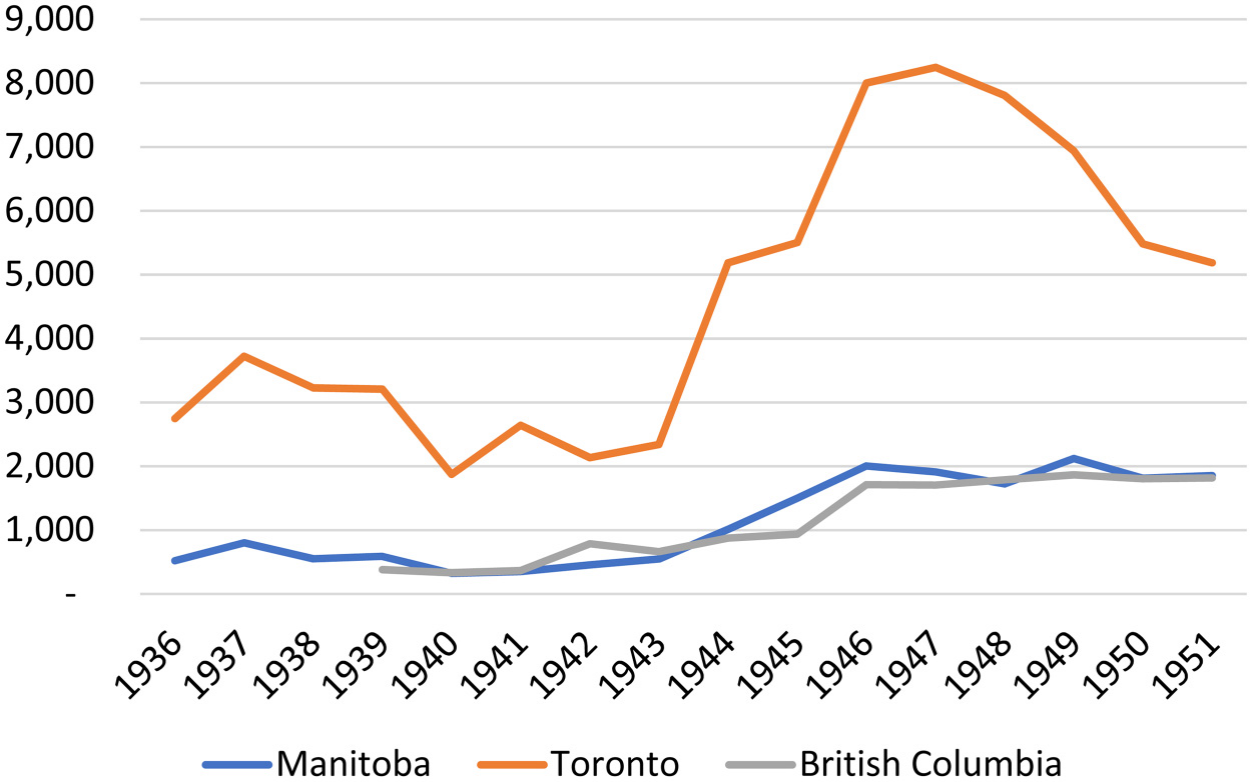
Enrollment in non-credit evening courses, provincial universities, 1936–1951.

Source: Annual Reports of the Presidents of the universities.

Smith's first two programmatic interventions were focused on non-credit evening courses whose direct costs could be recovered through tuition fees charged to participants. He established an “Evening Institute” in 1935 and offered courses consisting of fifteen lectures delivered once a week in the fall and winter. In the same year, Smith instituted an “Accountancy Course” (non-credit evening courses offered in collaboration with the Institute of Chartered Accountants). Evening Institute enrollments were evenly divided between five categories of courses: literature, writing, and public speaking; European languages; history and current events; fine arts, photography, and interior decorating; and commerce, accounting, and mathematics. Figure 4 compares annual enrollments in the Evening Institute and the Accountancy Course with those in comparable evening courses offered by the universities of Toronto and British Columbia.

At the University of Manitoba, enrollments in non-credit evening courses were strongly gendered, with women constituting 62% of students in courses relating to the humanities and fine arts, but just 3% of students in the Accountancy Course. Figure 4 shows that the University of Manitoba enrolled a comparable number of students in non-credit evening courses as did the Department of University Extension at the University of British Columbia. The University of Toronto, whose non-credit evening courses began in the early 1920s, was in a larger city and enrolled substantially more students.

The Evening Institute and Accountancy Course served adult learners in Winnipeg. Following the launch of the Evening Institute, Smith reflected:

We are still confronted with the problem of extending our educational services to the rural communities of the Province. Extension work of real value to those who register for it must consist of something more than the sporadic visits to communities of university professors whose fields of discussion are unrelated. I intend to invite my academic colleagues and members of the Board of Governors to consider, during the coming session, the best method, having regard to our straitened budgetary situation, of encouraging and assisting our fellow-citizens who live outside of Winnipeg to engage in systematic study in the fields of their choice (University of Manitoba, 1936, p. 1).

**Figure 5.**
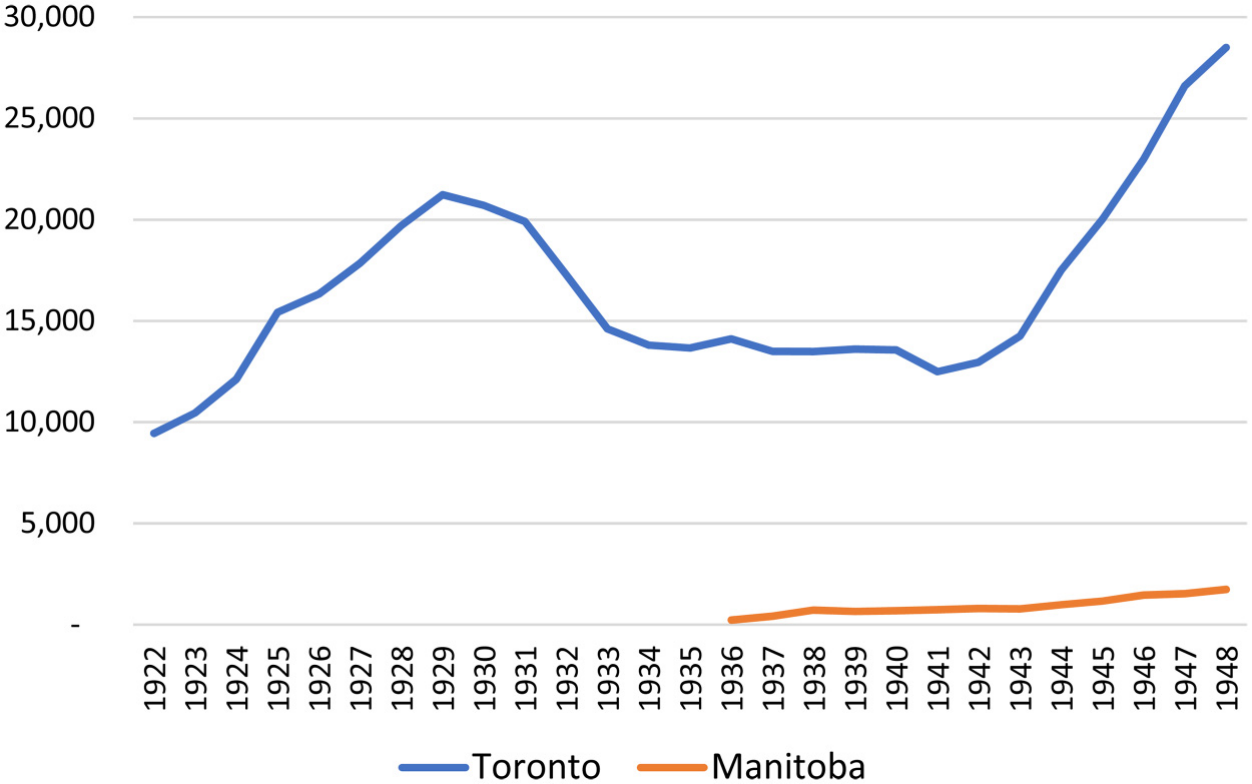
Musical examinations administered, provincial universities, 1922–1948.

Source: Annual Reports of the Presidents of the universities.

In subsequent years, Smith attracted funding from the Carnegie Corporation and Rockefeller Foundation to launch a series of adult educational initiatives in rural Manitoba. In 1936–37 and 1937–38, with funding from Carnegie, Miss Alice Johannsen visited dozens of communities to lecture and mount art exhibits in what Smith called a “remarkably fine programme of adult education in Fine Arts” (University of Manitoba, 1938, p. 3). In 1937, Smith established a “Department of Music Extension,” directed by Miss Eva Clare, whose work with adults outside of Winnipeg included broadcasting the “University Music Hour” and facilitating study groups around the province who met weekly to listen to, and discuss, the weekly radio broadcast (University of Manitoba, 1938). This department also developed syllabi and administered a system of exams in which students could achieve certificates for various levels of proficiency in musical theory, singing, and the playing of piano, violin, and organ. Figure 5 documents that while the University of Manitoba administered an annual average of over 900 such examinations between 1936 and 1948 (87% of which were taken by women and girls), such work represented only a fraction of the examinations administered across Canada by the University of Toronto, which had assumed responsibility for the Toronto Conservatory of Music in 1918.

**Figure 6.**
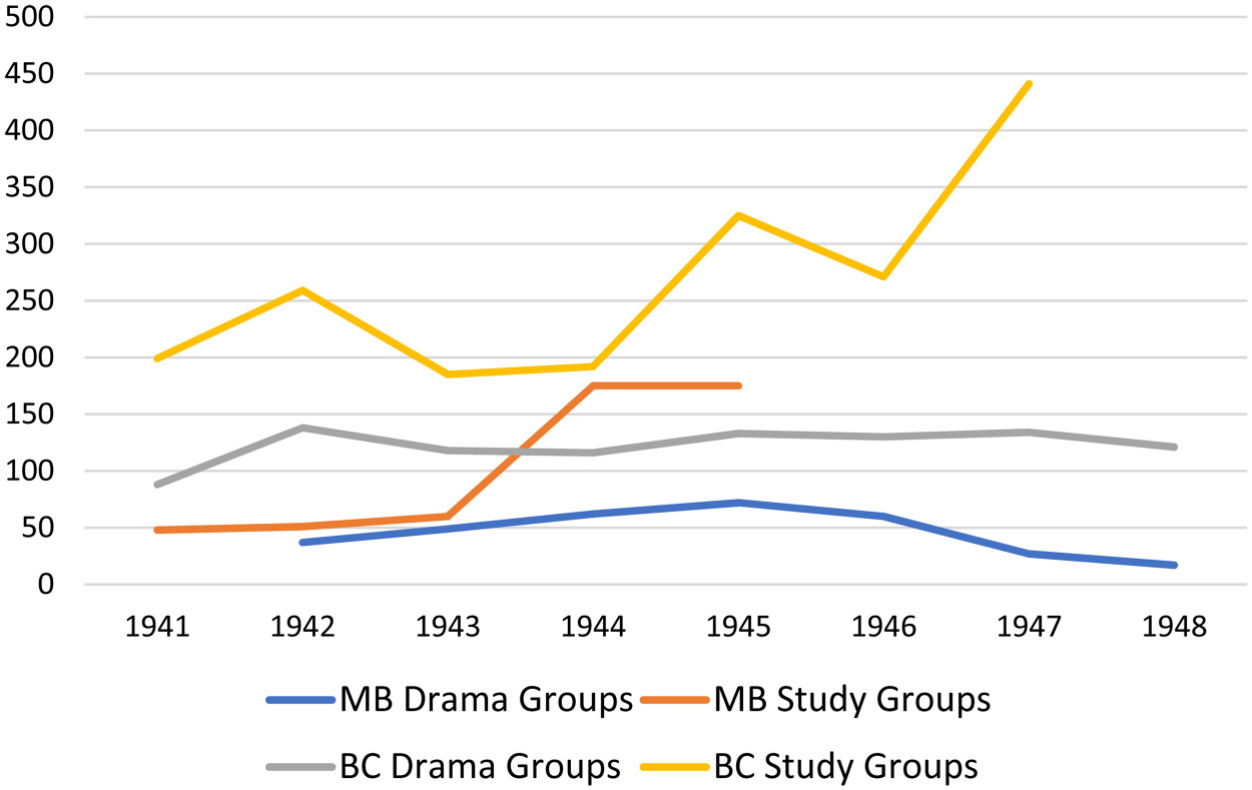
Number of study groups and drama groups organized, 1941–1948.

Source: Annual Reports of the Presidents of the universities.

**Figure 7.**
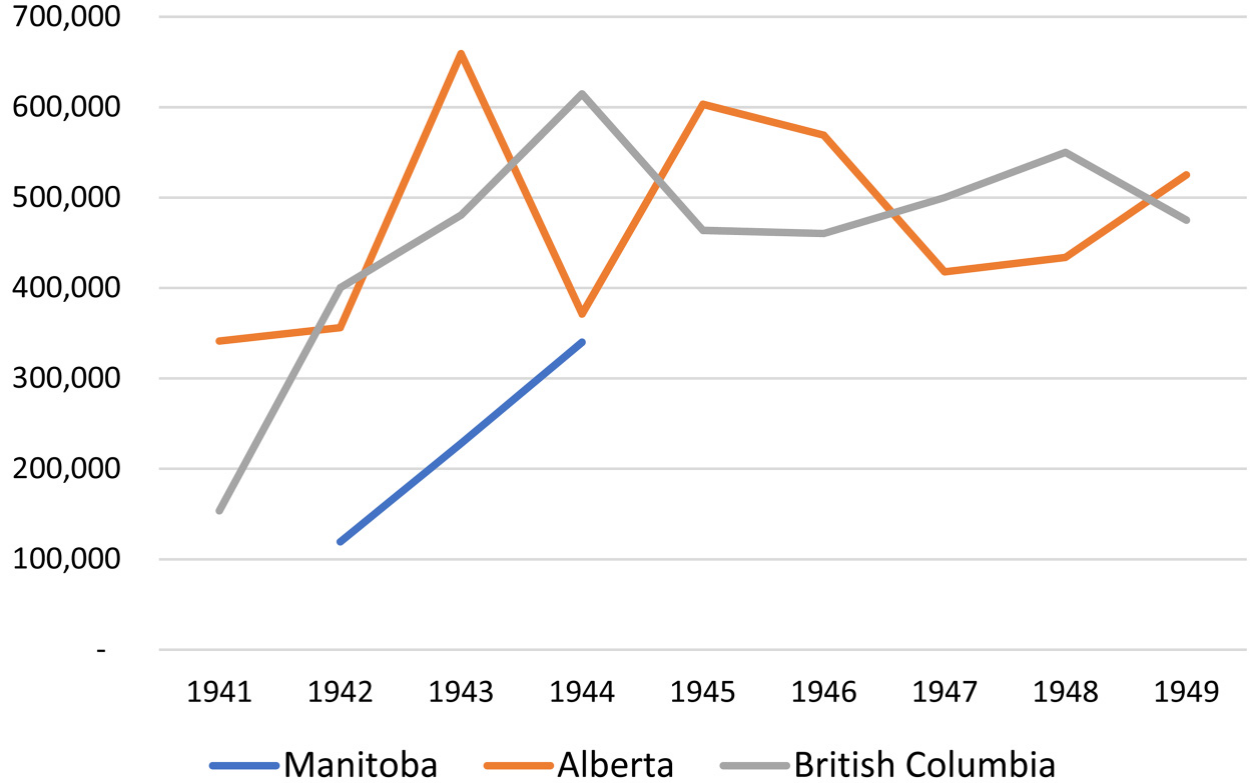
Aggregate annual attendance at monthly film circuits, 1941–1949.

Source: Annual Reports of the Presidents of the universities.

In 1939 and 1942, Smith obtained grants totaling $30,000 from Carnegie to support adult education work in rural Manitoba. These grants enabled the University of Manitoba to operate an Adult Education Office, directed by Watson Thomson and supported by up to seven members of staff between 1940 and 1945 (University of Manitoba, 1943). The work of the Adult Education Office focused primarily on organizing and facilitating small groups of adult learners across rural Manitoba to (1) engage in the study and discussion of print-based courses—mostly in the field of public affairs but also in diverse areas including art appreciation and child psychology—produced by universities across Western Canada and (2) engage in the reading or theatrical production of dramatic plays (note that work in drama was supported by an additional grant of over $6,000 from Rockefeller in 1942). In addition to activities that involved the facilitation of small groups of adult learners, the Adult Education Office also collaborated with the National Film Board to organize showings of educational films—often related to the war effort—across Manitoba. The typical program on these monthly circuits consisted of a feature film, a short educational film, and a short film of local or general interest. Figures 6 and 7 provide data about the scope of programming—at the University of Manitoba in comparison with the University of British Columbia and the University of Alberta—in the facilitation of study groups and drama groups and the showing of educational films.

Figures 6 and 7 illustrate the impact of institutional politics of adult education on the ability of universities to implement the Wisconsin idea of extension programming. The universities of Alberta and British Columbia, with extension departments funded by their respective provincial governments, sustained substantial levels of programming in small towns and rural areas throughout the 1940s. In contrast, the University of Manitoba, whose work in rural adult education was supported by short-term grants from Carnegie and Rockefeller, could not sustain such programming once the grant money had been spent. Following Thomson's resignation as Director of Adult Education in October 1944, the University of Manitoba appointed five acting directors, each of whom served for less than one year between 1944 and 1949: Stanley Rands, René Dussault, Mary Bishop, Barbara Chipman, and Dorothy Swancar. During the tenure of the three female acting directors, the Adult Education Office operated only eight months each year and had its staff restricted to an acting director and an office secretary earning a combined total of just $300 per month.

**Figure 8.**
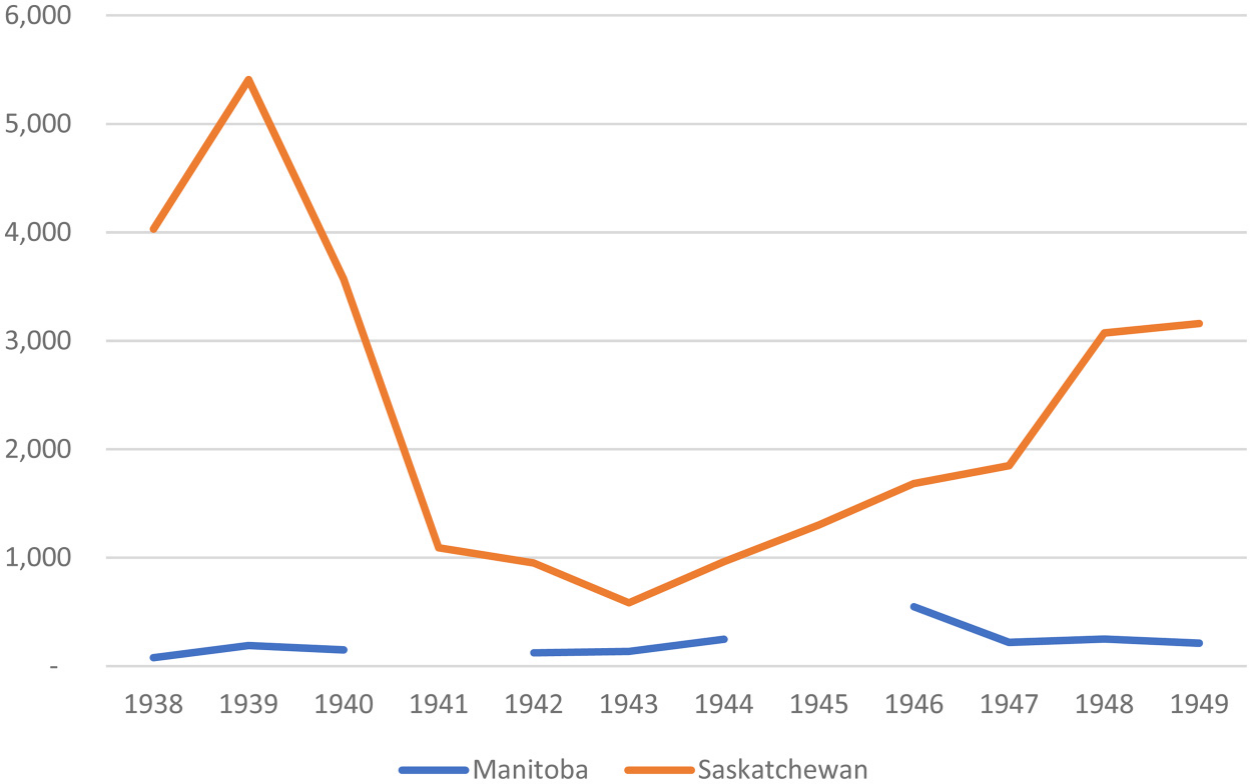
Enrollments in short courses in agriculture and home economics, 1938–1949.

Source: Annual Reports of the Presidents of the universities.

Data regarding work in agricultural extension provide a final indicator of the limited scope of activity related to the Wisconsin idea at the University of Manitoba in the 1930s and 1940s. Figure 8 reveals that while the University of Manitoba typically enrolled somewhere between 150 and 250 adults each year in short courses pertaining to agriculture and home economics, the number of enrollees in such courses was, on average, about ten times higher at the University of Saskatchewan. Taken together, Figures 4 through 8 illustrate that while the University of Manitoba significantly diversified and expanded its adult educational programming in the 1930s and early 1940s, such work was—apart from cost-recovery evening courses offered in Winnipeg—modest in comparison to that of other provincial universities.

Analyzing the politics of adult education at the University of Manitoba during this era shows that senior administrators deployed the Wisconsin idea as a rallying cry—something intended to inspire action or provoke a response, but something not necessarily integral to the strategic priorities of the institution. During Smith's decade as president, the University of Manitoba pursued extension work through recovering the costs of adult education programs through tuition fees and grants from American philanthropic agencies. The political logic behind his “serving our shareholders” rallying cry must be understood in the context of efforts to obtain more support from the provincial government for purposes unrelated to adult education. In 1939, Smith celebrated an increase in government funding:

It is not an exaggeration to state that the action of the Manitoba Legislature during its last session, in adding $50,000 to the University grant, was one of the most important events in the history of the institution…. Knowing my colleagues, I can faithfully pledge them to continue, with unabated zeal, to serve worthily the shareholders of the University, the people of Manitoba, and to justify in full this measure of increased support. (University of Manitoba, 1939, p. 1)

In the early 1940s, Smith continued his rhetorical campaign to build political capital through linking adult education with service to Manitobans. Following a report of the several adult educational initiatives launched during his tenure, Smith noted:

In several of my earlier Reports I expressed the hope that we could take the University to the country and that, on the cross-roads of Manitoba, people would speak of “our University.” I can now see the fulfillment, in very considerable measure, of that hope. I desire to express my deep-seated appreciation of the advice and assistance of my academic colleagues in this respect. They do not regard adult education as beneath their dignity; they regard it as complementary to instruction and research within the walls of the institution. (University of Manitoba, 1942, p. 4)

The next year, Smith claimed, “never before has the University taken to its shareholders—the citizens at large—so much of its scientific and cultural resources” (University of Manitoba, 1943, p. 7). In his final report as President, Smith reflected:

One of the projects developed during my Presidency, in which I take great pride, is adult education, both rural and urban. The project has served, yearly, thousands of the University's shareholders—the taxpayers of the Province… It is inconceivable to me that this undertaking, fraught with such advantages to the University and its shareholders, could be suspended or even curtailed after the expenditure of the grant of the Carnegie Corporation of New York. (University of Manitoba, 1944, p. 15)

Over the course of a decade, Smith built a diverse set of adult educational programs at the University of Manitoba. He did so without funding from the provincial government, by charging tuition fees for evening course participants in Winnipeg and by attracting grants for rural adult education programs. He regularly highlighted the results of these programs—measured by the number of Manitobans participating in them—to the provincial government as a means of building political capital. However, while modest increases to the budget of the university may have been partly attributable to the accumulation of such political capital, at no point during Smith's tenure did the provincial government provide funding for the University of Manitoba to engage in adult education activities.

In 1945, Albert Trueman became the third President of the University of Manitoba, serving for three years before leaving in 1948 to lead the University of New Brunswick. In his three annual reports as president, Trueman mentioned adult education only twice. In his first report, he stated, “It will be necessary, without doubt, to find means of continuing work in Adult Education, although I prefer to call it University Extension” (University of Manitoba, 1946, p. 5). In his second report, his only reference to adult education was to state, among a list of institutional priorities, that the university should “press for the establishment of a University Department of Extension” (University of Manitoba, 1947, p. 8). In his final annual report, Trueman did not mention adult education or extension. This did not mean that adult educational work had disappeared. Indeed, while the programs and staff of the Adult Education Office had shrunk substantially, participation increased substantially in the Evening Institute and Accountancy Course and in local exams in music.

The fact that Trueman barely mentioned adult education in his annual reports is notable because he served, from 1945 through 1947, as Chairman of the Manitoba Royal Commission on Adult Education. The provincial government asked that Commission:

To investigate the work now being carried on in the whole field of adult education in the Province of Manitoba, to advise and make recommendations to the Government of Manitoba on the co-ordination of such work in order to eliminate all overlapping, duplication, and conflict, to advise as to whether the whole field is now being adequately covered and if not as to what steps should be taken to cover that portion which is not. (Trueman, 1947, p. 6)

In its recommendations, the Commission was non-committal about the role of the University of Manitoba, stating that while the university was “an educational institution of major significance for adult education,” it was also “an autonomous body which must assume the responsibility for deciding what part it will play” (Trueman, 1947, p. 12). The Commission made one concrete recommendation (“the present administrative machinery of faculty committees should be replaced by a department of extension”) and one vague suggestion (“the university ought to exercise important influence through representation on councils or committees set up for the coordination of the educational efforts of voluntary and government agencies”) (Trueman, 1947, p. 12).

While the Manitoba Royal Commission on Adult Education did not set a direction for the University of Manitoba, it did lead the provincial government to commit funding to the university for purposes of coordinating adult education across the province. That commitment was made in a letter from C. Rhodes Smith (Provincial Minister of Education) to University of Manitoba President, Albert Gillson, on July 19 1949. Smith (1949) began his letter by stating that his government had “come to a decision on the general policy which it desires to follow in connection with Adult Education”. That policy was that publicly funded adult education should be non-partisan and should be conducted “with a view to providing the most all round educational facilities for our adult population” (Smith, 1949, p. 1). Smith stated that the “overall co-ordinating effort in adult education” should be “under the jurisdiction of the University of Manitoba” (Smith, 1949, p. 1). Smith explicitly stated that the university should not establish a “complex organization” to deliver adult educational programming; rather, Smith (1949) argued:

The program set up under the University should utilize the experience and interest of the many organizations which are now doing work in the adult education field. The work of all such organizations should be encouraged with a view to making each organization's work in its own field as broadly effective as possible. (pp. 1–2)

The University of Manitoba, according to the mandate outlined in Smith's letter, would be a coordinator rather than a provider of adult educational programming. Smith confirmed that the provincial government had committed about $7,000 for this “Adult Education Program” and that a key priority for the university should be the hiring of a director who would “work in conjunction with committees chosen partly from the University staff and partly from various organizations already at work in the adult education field” (Smith, 1949, pp. 3–4).

Stuart Tweedie became the first Director of the Department of University Extension and Adult Education on November 14 1949. His appointment satisfied the expectation of the mandate letter from the Minister of Education, but it did not expunge the institutional culture of indifference toward adult education that had developed since Sidney Smith's departure. Tweedie arrived at a “department” with no staff and inadequate office space. In his first report as Director, Tweedie described obtaining a secretary by arranging for the transfer of a woman from the university's typing pool and claimed, “it will not be possible to establish a smoothly running office in the present cramped accommodation” (Tweedie, 1949, p. 1). These conditions continued for years, with Tweedie reporting hiring his first member of staff in September 1951 and working with an annual grant of $16,000 from the provincial government (Tweedie, 1968). While the University of Manitoba had institutionalized extension through creating a department, it had not embraced a zeal for adult education.

## Explaining the Distinctive Politics of Adult Education at the University of Manitoba

The university extension movement of the late 1800s and early 1900s had a profound effect on the provincial universities of Ontario, Saskatchewan, Alberta, and British Columbia. The University of Toronto embraced the public lecture movement and later organized large numbers of evening courses and musical examinations. Universities in the three westernmost provinces embraced the Wisconsin idea, providing an impressive array of educational services to populations spread over vast distances. Why then, amid all this fervor for university extension, were leaders at the University of Manitoba unable to convince their provincial government to provide funding for adult education until a royal commission goaded them to do so in 1949? To answer this question, this section compares official statistics from five provinces to isolate the factors that distinguished the political economy of Manitoba.

**Figure 9.**
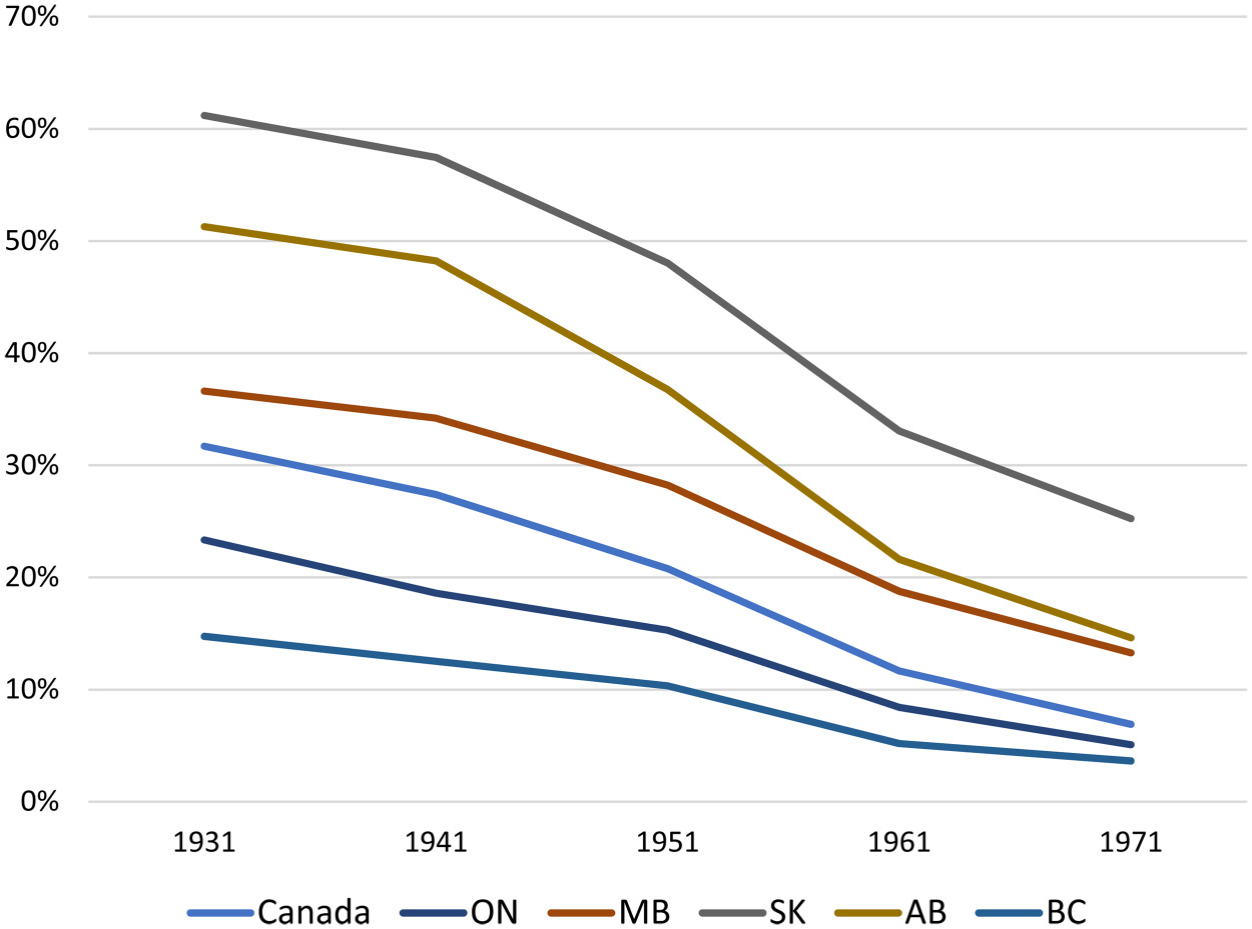
Proportion of the population living on farm, by province, 1931–1971.

Source: Statistics Canada, 1983, Tables A2–14, M1–11.

The fundamental transformation that all five provinces experienced over the course of the twentieth century was the movement of people away from rural livelihoods rooted in primary commodity production and toward urban livelihoods rooted in wage labor. Figure 9 shows that Manitoba evolved in a manner parallel to the Canadian average, while Saskatchewan and Alberta had much larger on-farm populations. Ontario and British Columbia had much smaller on-farm populations, since industrialization and urbanization started earlier in Ontario than elsewhere and since large numbers of people in British Columbia worked in non-agricultural rural commodity production (i.e., logging, mining, and fishing).

Figure 9 helps explain the prominence of the Wisconsin idea in Saskatchewan and Alberta, as well as the popularity of evening classes and extension lectures in Ontario and British Columbia, but it does not explain why the University of Manitoba was alone amongst provincial universities in being unable to secure public support for extension work.

**Figure 10.**
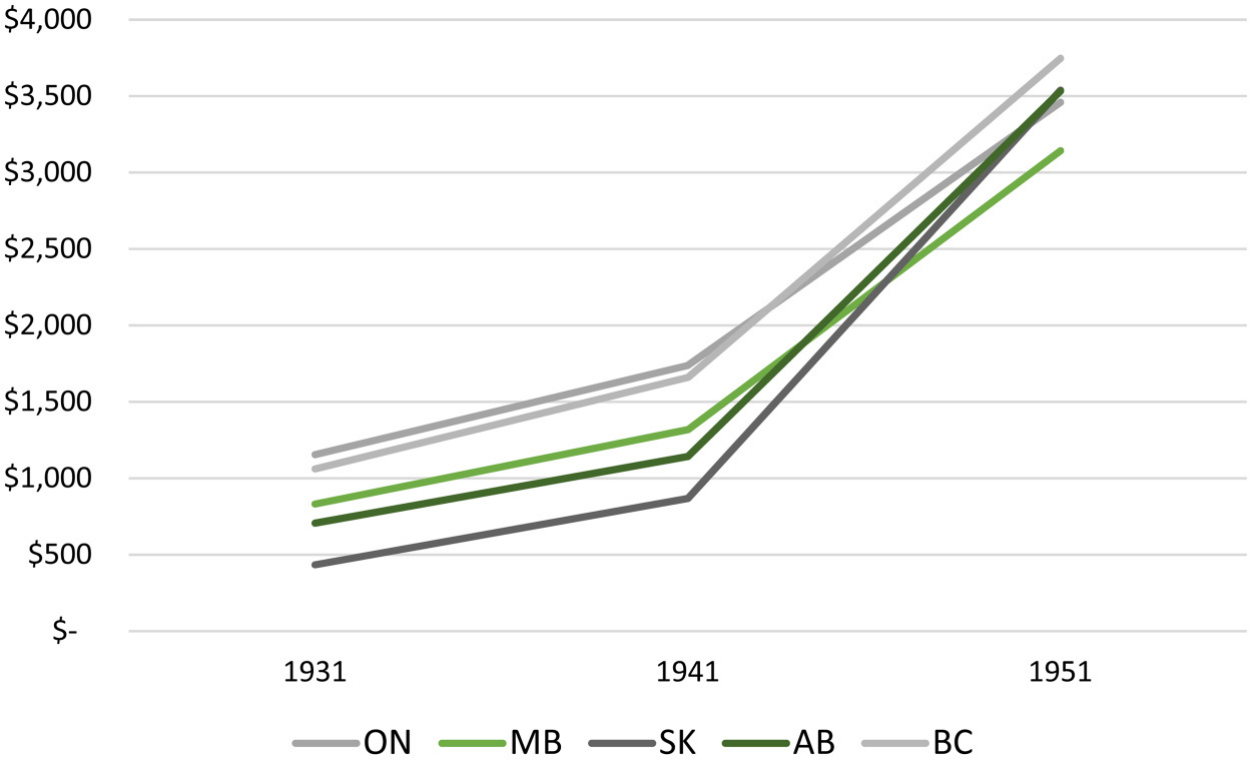
Average income of those in the labor force, by province, 1931–1951.

Source: Statistics Canada, 1983, Tables D512–521, F91–102.

Explaining the politics of adult education at the University of Manitoba requires an understanding of the approach of provincial governments toward taxation and public spending. The tax base of provincial governments depends upon income earned by the population of the province. Figure 10 demonstrates that average personal incomes in Manitoba—in comparison to those in other provinces—were about average in 1931 and 1941, while they lagged those other provinces by 1951. As such, the tax base of the Manitoba government was not dramatically different from that of the other provinces in the 1930s and 1940s.

**Figure 11.**
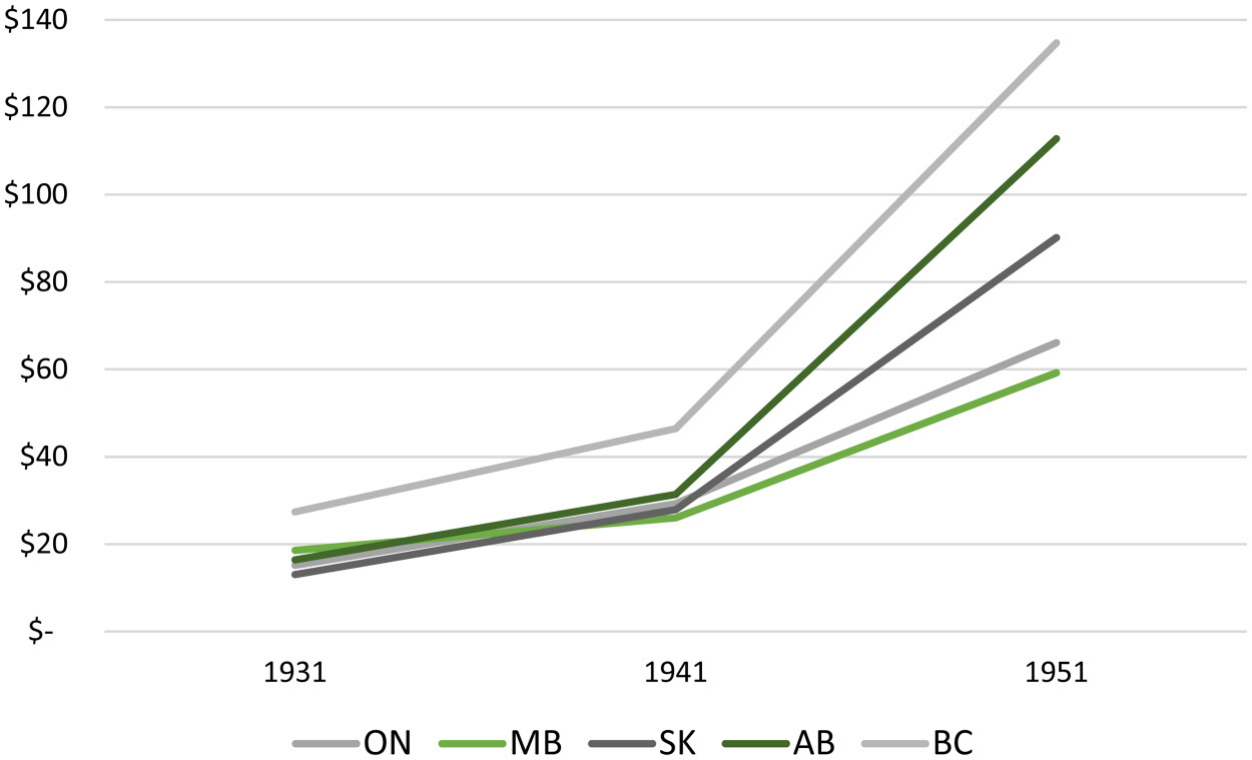
Per capita revenue of provincial governments, 1931–1951.

Source: Statistics Canada, 1983, Tables A2–14, H124–135.

What distinguished Manitoba more significantly from the other provinces in this era was the lack of willingness or ability of its provincial government to gather tax revenues. Figure 11 shows that a significant gap emerged, in per capita government revenues, between Manitoba and the westernmost provinces in the 1930s and 1940s.

By 1941, the government of Manitoba collected less revenue per capita than any these provinces. By 1951, per capita revenues of the Manitoba government were outpaced by 50% by the government of Saskatchewan and by over 100% by the government of British Columbia. Figure 11 shows a trend to fiscal conservatism in the provincial government of Manitoba—a hesitancy to collect taxes to create the foundation for spending on public goods and services such as higher education.

**Figure 12.**
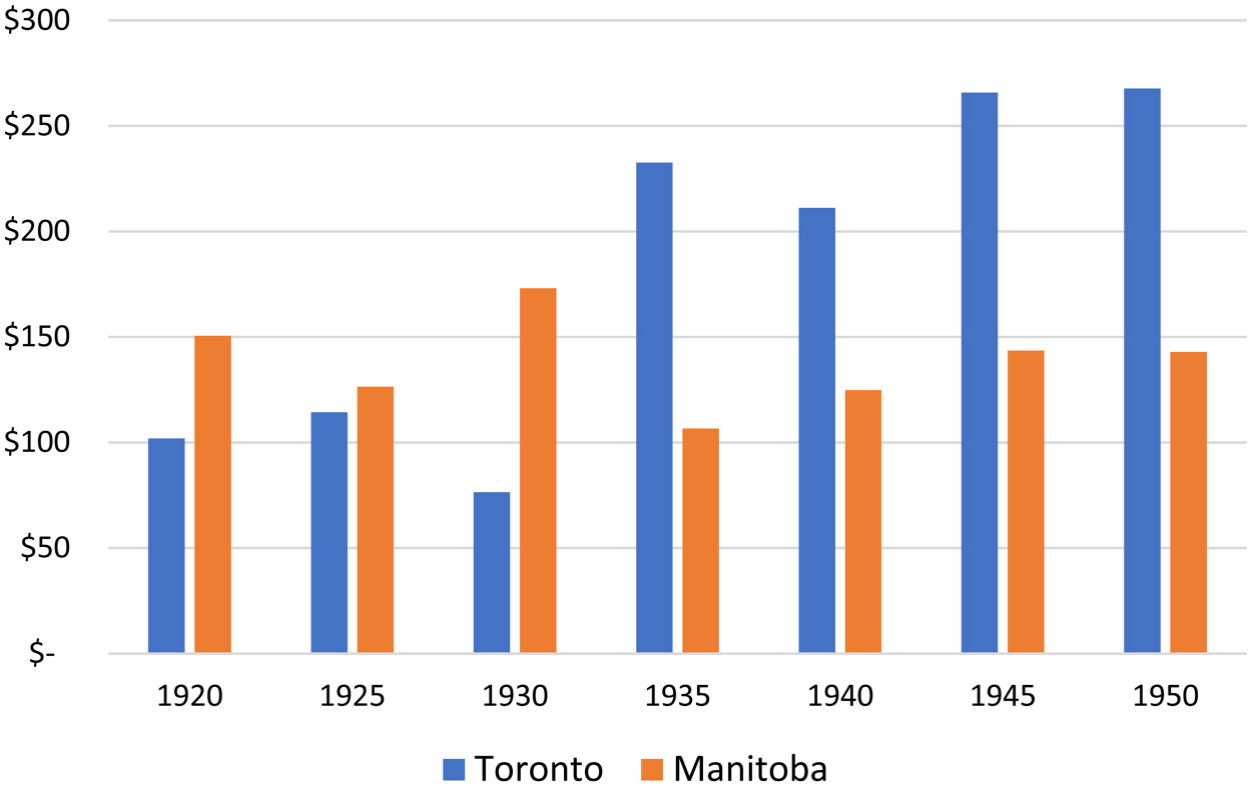
Provincial government grants per full-time student, 1920–1950.

Sources: Manitoba Department of Education, 1930, 1940, 1949; Annual Reports of the Board of Governors of the University of Toronto.

Growing fiscal conservatism within the Manitoba government was linked to the overall budgetary fortunes of its provincial university. For over a decade after the University of Manitoba became a provincial university in 1917, the government of Manitoba supported the institution with more funding, on a per full-time student basis, than the government of Ontario granted to the University of Toronto (see Figure 12). However, in the 1930s and 1940s, government grants to the provincial universities increased in Ontario and decreased in Manitoba. One result of fiscal conservatism in the Manitoba government, then, was the relative underfunding of its provincial university.

Another result of such fiscal conservatism was the lack of willingness of the Manitoba government to support extension work as did provincial governments to the west. The relatively large number of full-time students at the University of Manitoba (see Figures 1 and 2) likely enabled members of the provincial government to believe that their support of higher education was sufficient and that there was no need (or no politically salient demand) for them to support university extension work. In short, a relatively large student body combined with fiscal conservatism and the relative stagnation of incomes in Manitoba reduced the importance of extension work in the eyes of decision makers with the provincial government.

## Conclusion

Both waves of the university extension movement were experienced differently at the University of Manitoba than at other provincial universities in central and western Canada. The public lecture movement began in earnest in 1894 at the University of Toronto and later became a significant feature of the role of the provincial universities in the lives of people in Ontario, Alberta, and British Columbia. The Wisconsin idea took off in Canada with the establishment of departments of extension at the University of Saskatchewan in 1910 and the University of Alberta in 1912. Those departments—focused on agriculture and home economics in Saskatchewan and the cultural enrichment of rural communities in Alberta—became major institutions of adult education in their respective provinces. In contrast, neither the public lecture movement nor the Wisconsin idea was sustained at the University of Manitoba, whose provincial government only began funding a Department of University Extension and Adult Education in 1949.

This article has described the distinctive politics of adult education at the University of Manitoba by comparing its engagement in the university extension movement with that of four other provincial universities in Canada. It has shown that while institutional leaders mobilized the rhetoric and the practice of adult education for political purposes—ranging from that of President MacLean's sacrificial lamb of extension lectures to that of President Smith's rallying cry of the Wisconsin idea of service—they at no point were able to convince decision makers in the provincial government to fund adult educational work through the university.

Rather than seek an explanation of the inability of the University of Manitoba to institutionalize extension work in the machinations of conservative politicians or the shortcomings of institutional leaders, this study has grounded its analysis in distinctive characteristics of the province of Manitoba in the first half of the twentieth century: the fact that the University of Manitoba enrolled more full-time students per capita than did any other university; the fact that personal incomes grew more slowly in Manitoba than they did in any other province; and the trend toward fiscal conservatism in both overall provincial government revenues and the funding of the provincial university. In a socio-political context in which provincial decision makers were hesitant to collect and spend money, the fact that the University of Manitoba served relatively large numbers of degree-credit students made university extension less politically attractive than it was elsewhere.

For readers with an interest in the history of adult education, this article has narrated the politics of the extension movement at what was one of Canada's largest universities. For readers interested in the contemporary politics of adult education, this article has highlighted two key lessons. First, the evolution of institutional structures and programs reflects the political strategies of university administrators engaged in managing relationships with government. Second, those political strategies are influenced by socio-economic and institutional developments that are not directly related to adult education. These lessons—elaborated here through a historical study from Canada—are significant for contemporary scholars and practitioners of adult education elsewhere.
